# Antibiotic Resistance Genes in Dust from Kindergarten Environments: A Systematic Review of Occurrence, Diversity, Determinants, and Exposure Implications

**DOI:** 10.3390/ijerph23081036

**Published:** 2026-08-09

**Authors:** Prasert Makkaew, Apirak Bumyut, Ni Luh Ayu Megasari, Nopadol Precha

**Affiliations:** 1 Department of Environmental Health and Technology, School of Public Health, Walailak University, Thasala, Nakhon Si Thammarat 80160, Thailand; mprasert@wu.ac.th (P.M.); apirak.bu@wu.ac.th (A.B.); 2 Immunology Program, Postgraduate School, Universitas Airlangga, Surabaya 60286, East Java, Indonesia; ni.luh.ayu@pasca.unair.ac.id

**Keywords:** child, drug resistance, dust, integrons, preschool

## Abstract

**Highlights:**

**Public health relevance—How does this work relate to a public health issue?**

- Across the four included studies, kindergarten dust consistently harbored clinically important antibiotic resistance genes (ARGs) including last-resort resistance determinants *mecA*, *vanA*, and *bla_NDM_* across geographically diverse settings in China, Hong Kong, and Norway.
- Antibiotic-resistant bacteria carrying resistance markers concordant with kindergarten AC-filter dust profiles were detected in urine samples from children attending those same facilities.

**Public health significance—Why is this work of significance to public health?**

- Class 1 integron-integrase genes (*intI1*) co-occurred with ARGs in three studies, and pathogen-derived extracellular vesicles enriched with multidrug efflux genes were identified in kindergarten dust.
- This systematic review identified winter seasons and urban location are associated with increased ARG abundance in kindergarten dust.

**Public health implications—What are the key implications or messages for practitioners, policy makers and/or researchers in public health?**

- Kindergartens warrant prioritization as a candidate setting for future environmental AMR surveillance research, with evaluation of candidate dust-monitoring genes such as *sul1*, *sul2*, *intI1*, *mecA*, *tetQ*, and *ermB*.
- Prospective cohort studies and intervention trials are needed to evaluate links between kindergarten dust resistomes, children’s AMR outcomes, and the effectiveness of ventilation and cleaning strategies.

**Abstract:**

Kindergarten environments combine high microbial exposure with increased immunological vulnerability, yet antibiotic resistance genes (ARGs) in kindergarten dust remain poorly characterized. This systematic review synthesized evidence on the occurrence and potential health relevance of ARGs in kindergarten dust. Following PRISMA 2020 guidelines, PubMed, Scopus, and Web of Science were searched. Four studies from China, Hong Kong, and Norway (2018–2024) met the inclusion criteria. ARGs were detected in all kindergarten dust samples, indicating that dust is a consistent reservoir of antibiotic resistance determinants. A consensus resistome (classes detected in ≥2 studies) encompassed sulfonamide, macrolide–lincosamide–streptogramin B (MLSB), tetracycline, beta-lactam, aminoglycoside, and multidrug resistance genes; beta-lactam resistance genes were the only class reported in all four studies. Clinically important ARGs associated with last-resort antibiotics, including *mecA*, *vanA*, *bla_NDM_,* and *mcr-5*, were reported in three studies. Class 1 integron-integrase genes (*intI1*) frequently co-occurred with ARGs, suggesting potential horizontal gene transfer. Limited evidence indicated higher ARG abundance in urban and winter samples. One study reported antibiotic-resistant bacteria carrying resistance markers concordant with those in kindergarten dust in the urine of children attending the same facilities; however, this cross-sectional, single-site evidence is consistent with, but not sufficient to establish, a dust-to-child exposure pathway. The available evidence supports the plausibility that kindergarten dust may contribute to children’s exposure to ARGs and ARG-carrying bacteria, but current studies do not establish causal transmission from dust to child colonization or infection. Standardized monitoring and longitudinal studies are needed to assess health risks and guide mitigation strategies in early childhood educational settings.

## 1. Introduction

Antimicrobial resistance (AMR) represents one of the threats to global public health in the twenty-first century. The most comprehensive global burden analysis to date estimated that bacterial AMR was directly attributable to 1.27 million deaths and associated with approximately 4.95 million deaths worldwide in 2019, across 204 countries and 88 pathogen–drug combinations [1]. Particularly concerning is the disproportionate impact on young children, with one in five AMR-attributable deaths occurring in children under five years of age, frequently from infections that were previously treatable with first-line antibiotics [1]. Without accelerated intervention, AMR-attributable mortality is projected to rise to 1.91 million deaths annually by 2050 [2]. The World Health Organization has designated AMR as one of the top ten global public health threats facing humanity, and calls for a “One Health” approach that integrates human, animal, and environmental dimensions of resistance spread [3]. A critical step in addressing this challenge is understanding the transmission pathways of ARGs to vulnerable populations and resolving the evidence gaps that limit effective interventions.

The spread of antibiotic resistance genes (ARGs) extends well beyond the conventional reservoirs of resistance, including hospitals, livestock facilities, and wastewater treatment plants [4,5]. ARGs are now widely documented across environmental sources including soil, water, and ambient air, and are increasingly recognized as persistent constituents of the built indoor environment [6,7]. Indoor dust represents a complex environmental matrix containing particulate matter, biological material, and chemical pollutants, and serves as a major reservoir of microorganisms and ARGs in urban areas [6,7,8,9]. Global surveys of airborne ARGs have demonstrated their detection across 19 world cities, with relative abundances varying by nearly 100-fold between locations, and significant concentrations reported in indoor particulate matter from hospitals, offices, and residential environments [10,11]. Critically, ARGs identified in dust and indoor air are frequently associated with mobile genetic elements (MGEs), including integrons, plasmids, transposons, and insertion sequences, indicating their potential for horizontal gene transfer (HGT) between bacterial populations [12,13]. Accordingly, the detection of ARGs together with MGEs implies a greater potential for the spread of antibiotic resistance within microbial communities and across environments, substantially increasing the public health concern compared with the presence of ARGs alone.

Of all indoor environments, kindergartens and daycare centers occupy a uniquely important position in the AMR exposure landscape, for reasons that are both biological and behavioral. Schools are important environments where children may spend up to 10 h each day [14]. Dust in classrooms, floors, desks, Heating Ventilation and Air Conditioning (HVAC) systems, and other indoor surfaces may act as a reservoir for bacteria, antibiotic-resistant bacteria, antibiotic resistance genes, and mobile genetic elements [15,16,17,18]. Children aged one to six years represent a physiologically vulnerable group, because their respiratory, immune, and endocrine systems remain incompletely developed, rendering them more susceptible than adults to the health consequences of environmental microbial exposures [19,20,21]. Children are more susceptible to resistant bacteria due to their immune systems are not fully developed, and those in low-resource settings with limited access to health services [22,23]. Compounding this biological vulnerability is the behavioral profile of young children in group care settings. Concentrations of resuspended floor-dust bacteria have been reported to be 8–21 times higher in the infant breathing zone near the floor than in the adult breathing zone [24,25]. In addition, children frequently engage in hand-to-mouth behaviors and lack consistent hand hygiene practices, increasing the likelihood of ingesting or inhaling dust-associated microorganisms and their genetic components [26,27]. Exposure risk may be further intensified by the amount of time children spend in institutional childcare settings. In Norway, 91% of children aged 1–5 years attend kindergarten, and 95% spend more than 41 h per week there [15]. In China, children aged 3–6 years attend mandatory preschool for approximately 40 h per week [17]. The prolonged daily duration of exposure, combined with floor-level activity and intensive peer interactions, creates a sustained interface between children and the indoor dust resistome.

Despite the recognized vulnerability of young children and the documented ubiquity of ARGs in indoor environments, kindergartens have received disproportionately little systematic attention as sites of ARG exposure. Previous research has characterized ARGs in household dust [7], hospital air [11], urban outdoor particulate matter [28], and general workplaces [29], but these settings differ substantially from kindergartens in terms of occupant age, duration of exposure, floor-level activity, and the specific ventilation, hygiene, and building-use characteristics of childcare facilities. Individual studies conducted between 2018 and 2024 have begun to document ARGs in kindergarten dust across diverse geographic contexts, including outdoor impervious surface dust from urban China [17], extracellular vesicle (EV)-associated indoor dust fractions in China [18], air-conditioner filter dust from Hong Kong [16], and floor dust from Norway [15], but their findings have not yet been synthesized within a formal systematic review framework. Without the synthesis, it remains impossible to draw evidence-based conclusions about the consistency, magnitude, or public health significance of ARG exposure in kindergarten environments, and to identify which environmental, seasonal, and building-related factors are the most important modifiers of that exposure. This gap is particularly consequential given that the early childhood represents a critical and potentially irreversible period for immune system maturation and microbiome development [30,31].

This systematic review was therefore conducted to provide the first comprehensive synthesis of the evidence on ARGs in kindergarten dust and their implications for children’s health. Following the Preferred Reporting Items for Systematic Reviews and Meta-Analyses (PRISMA) 2020 guidelines, we systematically searched and critically appraised the available literature with five specific objectives: (1) to characterize the abundance, diversity, and distribution of ARGs detected in kindergarten dust across different countries, seasons, and sampling sites; (2) to identify the ARG classes and subtypes most consistently reported across studies; (3) to evaluate the co-occurrence of ARGs with mobile genetic elements and potential human pathogens; (4) to examine the environmental, seasonal, and building-related factors that modulate ARG abundance in kindergarten; and (5) to assess the available evidence concerning children’s exposure to dust-associated ARGs and the associated health risk. This review aims to provide an evidence base to inform the development of monitoring standards, hygiene guidelines, and targeted interventions to protect young children from ARG exposure in kindergarten environments.

## 2. Materials and Methods

### 2.1. Study Protocol

**Figure 1:**
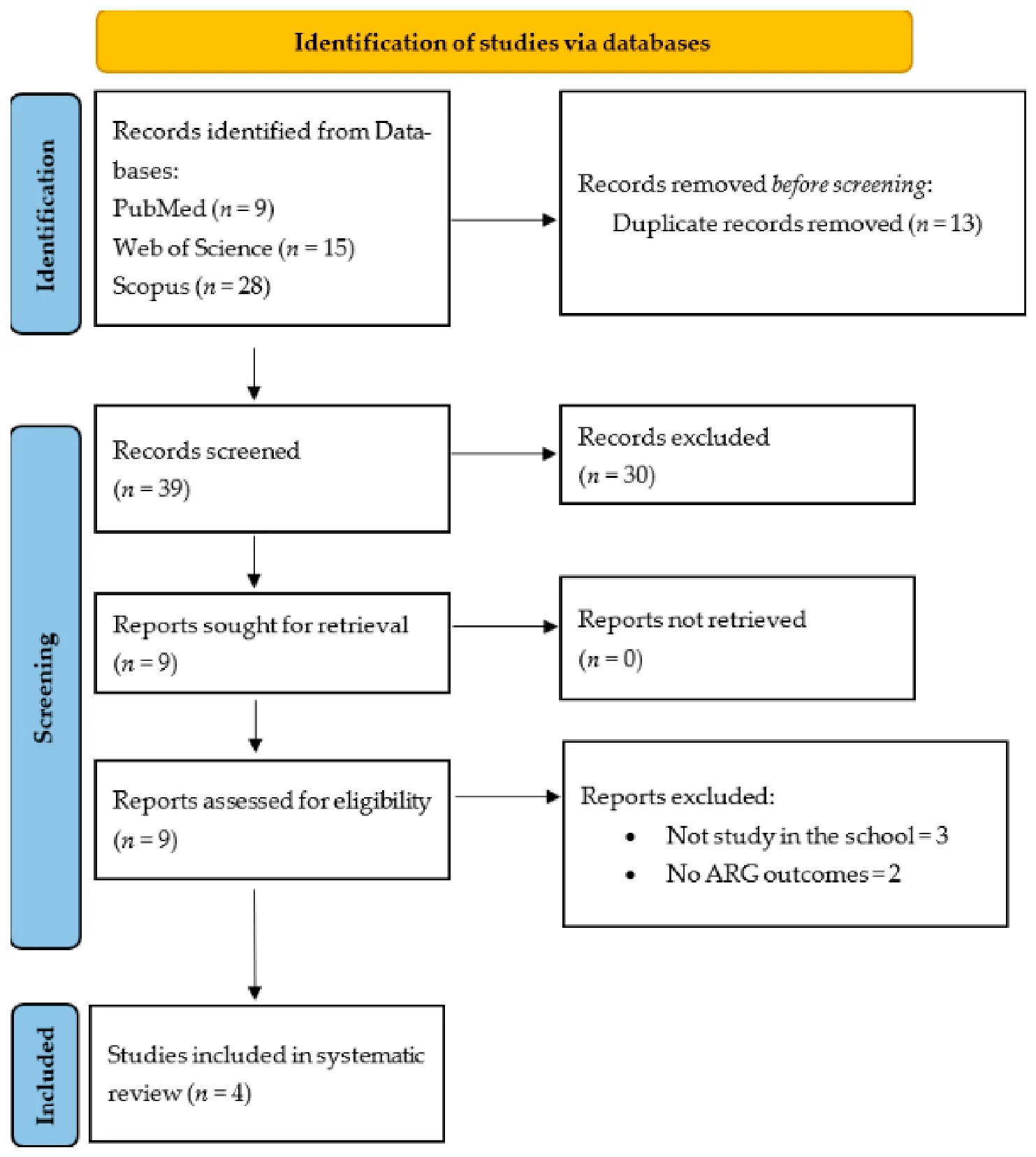
The PRISMA flow diagram of the study search of databases.

This study was conducted as a systematic review following the Preferred Reporting Items for Systematic Reviews and Meta-Analyses (PRISMA) 2020 guidelines [32]. The review was designed to identify, appraise, and synthesize primary studies that have examined antibiotic resistance genes (ARGs) in dust collected from kindergarten environments attended by children aged 1–6 years. No meta-analysis was performed due to the substantial methodological heterogeneity among the included studies; findings were therefore synthesized narratively. The review protocol was prospectively developed prior to database searching, with predefined objectives, eligibility criteria, search strategy, data extraction items, and a risk of bias assessment approach. In accordance with the PRISMA 2020 requirements for registration and protocol availability, this review was not registered in a public systematic review registry such as PROSPERO, and the prospectively developed protocol was not published in, or deposited with, an external repository. The completed PRISMA 2020 checklist is provided in Supplementary Table S1, and the study selection process is reported using the PRISMA 2020 flow diagram (Figure 1).

### 2.2. Eligibility Criteria

Studies were assessed for eligibility against predefined inclusion and exclusion criteria developed using the Population, Exposure, Comparator, and Outcome (PECO) framework. The population of interest was children aged 1–6 years attending kindergarten, preschool, or daycare environments. The exposure of interest was antibiotic resistance genes (ARGs) in dust collected from within or immediately surrounding those kindergarten premises. No comparator was required for inclusion, as the primary aim was characterization of ARG occurrence rather than comparative effect estimation. The primary outcome was the detection and/or quantification of at least one ARG class or subtype in dust samples using a validated molecular method. The full inclusion and exclusion criteria are presented in Table 1.

### 2.3. Information Sources and Search Strategy

A systematic search of three major electronic bibliographic databases was conducted: PubMed, Scopus, and Web of Science. These databases were selected for their comprehensive indexing of peer-reviewed literature in the fields of environmental health, microbiology, public health, and indoor air quality. The search was conducted in March 2026, with no restriction on publication start date, and was limited to articles published in English. No grey literature, conference proceedings, or non-peer-reviewed sources were searched.

Search strings were constructed using a combination of Medical Subject Headings (MeSH) terms where available and free-text keywords covering three conceptual domains: (i) the target setting, including kindergarten and equivalent early childhood educational environments; (ii) antibiotic resistance, encompassing antibiotic resistance genes (ARGs), antimicrobial resistance (AMR), and the resistome; and (iii) environmental matrix, including dust, indoor air, settled dust, floor dust, and AC-filter dust. Boolean operators (AND, OR) were used to combine terms within and across domains. The complete search strings used for each database are presented in Table S1.

### 2.4. Study Selection

All records retrieved from the three database searches were imported into a reference management software (EndNote X9; Clarivate Analytics, Philadelphia, PA, USA) and deduplicated automatically, followed by manual verification to ensure complete removal of duplicate entries. The study selection process was conducted in two sequential stages by three independent reviewers (hereafter referred to as Reviewer 1, Reviewer 2, and Reviewer 3), with disagreements resolved through discussion and consensus at each stage. Where consensus could not be reached between any two reviewers, the third reviewer served as the final arbitrator. In the first stage, titles and abstracts of all deduplicated records were independently screened by all three reviewers against the predefined eligibility criteria. Each record was assessed by at least two reviewers independently and in parallel, without knowledge of the other reviewer’s decision prior to comparison. Records were excluded at this stage if the title and abstract provided sufficient information to confirm that the study did not meet one or more inclusion criteria. Records for which eligibility could not be determined from the title and abstract alone were retained for full-text assessment. Inter-rater agreement at the title and abstract screening stage was κ = 0.71, indicating substantial agreement. In the second stage, full-text articles of all records that passed title and abstract screening were independently retrieved and assessed for eligibility in full by all three reviewers. Each full-text article was independently evaluated by at least two reviewers prior to comparison of decisions. The specific reason for exclusion was recorded for each full-text article that did not meet the eligibility criteria, using a standardized exclusion reason coding scheme aligned with the predefined inclusion and exclusion criteria (Table 1). Studies meeting all eligibility criteria following full-text assessment were included in the final qualitative synthesis. The complete study selection process, including the number of records identified, deduplicated, screened, retrieved, assessed, excluded with reasons, and included at each stage, is presented as PRISMA 2020 flow diagram.

### 2.5. Data Extraction

Data were extracted from all four included studies using a standardized extraction form developed specifically for this review. Data extraction was performed by a single reviewer and verified by cross-checking against the original full-text articles. Where data were available in Supplementary Materials, these were accessed and extracted alongside main manuscript data. The data extraction form captured the following categories of information; study identification, study design, setting characteristics, sampling period, sampling matrix and collection method, ARG detection method and annotation database, ARG outcomes including the classes and subtypes detected and the normalization strategy used, mobile genetic element (MGE) outcomes, microbial community characterization, reported environmental factors associated with ARG abundance, and any available human exposure or child biomonitoring data. For ARG outcomes specifically, all resistance gene classes and subtypes reported in the primary studies were extracted and tabulated to enable cross-study comparison. To ensure consistent cross-study terminology, ARG classes were categorized a priori as: (i) universally reported classes, detected in all four included studies; (ii) consensus classes, detected in at least two included studies; and (iii) the study-specific expanded resistome, comprising classes or subtypes reported only in individual high-resolution High-throughput qPCR (HT-qPCR) or shotgun-metagenomic studies.

### 2.6. Risk of Bias Assessment

The methodological quality and risk of bias of each included study was assessed using an adapted version of the Newcastle–Ottawa Scale (NOS) for observational studies. The original NOS was developed for epidemiological cohort and case–control studies and awards a maximum of nine stars across three domains: selection, comparability, and outcome. For the purposes of this review, the NOS was modified to reflect the cross-sectional, environmental exposure design of kindergarten dust resistome studies, resulting in an adapted eight-item instrument covering all three domains. Each item was scored as either met (one star awarded, ★) or not met (zero stars, ☆), yielding a maximum score of eight stars. Studies achieving six or more stars were classified as low risk of bias; those achieving four to five stars as moderate risk of bias; and those achieving three or fewer stars as high risk of bias. Because the four included studies had heterogeneous objectives, the adapted assessment tool was applied only to two inferential targets: (i) reliable detection of antibiotic resistance genes (ARGs) and (ii) estimation of ARG abundance. It was not intended to evaluate inference regarding environmental determinants of ARG occurrence or child health risks, for which the included observational descriptive studies provide inherently limited evidence. Consequently, a study may be judged as having a low risk of bias for ARG detection while remaining uninformative for causal or health-risk inference. The adapted eight-item instrument was developed specifically for this review and has not undergone formal psychometric validation. Accordingly, the resulting scores should be interpreted as a transparent, structured appraisal rather than a validated measure of methodological quality. The rationale for each adapted domain and its correspondence with the original Newcastle–Ottawa Scale (NOS) are presented in Supplementary Tables S2 and S3.

### 2.7. Synthesis Approach

A narrative synthesis approach was adopted for this review, as the substantial methodological heterogeneity across the four included studies, encompassing differences in ARG detection platforms, abundance normalization strategies, sampling matrix, study designs, and geographic settings, precluded any meaningful quantitative meta-analysis. Pooling of ARG abundance estimates across studies was not performed because the normalization denominators used (copies per gram of dust, copies per 16S rRNA gene copy, copies per cell, and relative proportions of metagenomic reads) are fundamentally incompatible and cannot be converted to a common metric without introducing substantial assumptions.

The narrative synthesis was structured around six thematic domains derived from the predefined review objectives and data extraction categories: (i) the distribution and diversity of ARG classes and subtypes across studies; (ii) the co-occurrence of ARGs with mobile genetic elements (MGEs) and its implications for horizontal gene transfer; (iii) environmental factors associated with ARG abundance including season, urbanization, and ventilation type; (iv) source attribution of ARGs in kindergarten dust; (v) children’s exposure potential and available human biomonitoring evidence; and (vi) methodological heterogeneity and its implications for evidence synthesis. Where findings from two or more studies converged on the same direction or conclusion, these were characterized as consistent findings. Where findings diverged or were available from only a single study, these were explicitly identified as preliminary or hypothesis-generating observations requiring replication.

All included studies were primary observational investigations. No randomized controlled trials, intervention studies, or experimental studies were identified during the search. Consequently, no effect size estimates, confidence intervals, or heterogeneity statistics (I^2^, Cochran’s Q) were calculated. Because no quantitative synthesis was undertaken and only a small number of studies met the inclusion criteria, formal statistical assessment of reporting bias arising from missing results was not feasible; the potential for reporting and publication bias was instead considered narratively. For the same reasons, the certainty of the body of evidence was not graded using a formal instrument such as GRADE. Instead, confidence in each synthesized finding was appraised narratively based on the consistency of results across studies, the number of contributing studies, and their risk-of-bias ratings, with findings supported by only a single study explicitly identified as preliminary or hypothesis-generating.

### 2.8. Ethical Considerations

This systematic review is based exclusively on previously published, publicly available primary studies and did not involve the collection of new primary data from human participants, animals, or the environment. Accordingly, no ethical approval was required for the conduct of this review. All included studies cited in this review reported their own ethical approval and participant consent procedures in their respective publications, and these are not repeated here. No personally identifiable data from study participants were accessed, extracted, or reported in this review.

## 3. Results

### 3.1. Search Results

A total of 52 articles were identified through database searching, including PubMed (*n* = 9), Scopus (*n* = 28), and Web of Science (*n* = 15) (Figure 1). After the removal of 13 duplicate articles, 39 unique articles remained for title and abstract screening. During the screening process, 30 articles were excluded because they did not meet the predefined inclusion criteria, primarily due to irrelevant study settings, absence of dust samples, or lack of antibiotic resistance gene (ARG) outcomes. Nine reports were sought for retrieval, and all full-text articles were successfully obtained for eligibility assessment. The PRISMA checklist guidelines were provided in the Supplementary Material File. The PRISMA flow states that five full-text articles were excluded: three studies were not conducted in school environments, and two studies did not report ARG-related outcomes. Consequently, four articles [15,16,17,18] were included in the final qualitative synthesis of this systematic review.

### 3.2. Study Characteristics

A total of four eligible studies identified through systematic search matched with all defined inclusion criteria. All four studies investigated antibiotic resistance genes (ARGs) in dust collected from kindergartens attended by children aged 1–6 years. Three studies were conducted in China (including one specifically in Hong Kong) [16,17,18], and one study was conducted in Norway [15]. Publication years ranged from 2018 to 2024, indicating a relatively recent and emerging body of evidence. Among the four included studies, most employed a cross-sectional design (75.0%, n = 3), while one study (25.0%) used a longitudinal design with five repeated sampling rounds conducted over eleven months in a newly opened kindergarten (Table 2). Most studies collected samples from indoor environments (75.0%, n = 3), whereas one study (25.0%) focused on the outdoor environment of a kindergarten. Among the sampling sites, air-conditioning (AC) filter dust and settled floor dust were the most frequently investigated (50.0%, n = 2), while the remaining sampling sites were each represented in 25.0% of studies. Of the four studies, three collected samples using vacuum-based methods, while one study used a passive sampling method. One study in China (2024) investigated 59 kindergartens across two seasons, generating 118 samples, representing the largest dataset in this review. Another study in China (2020) collected air-conditioning (AC) filter dust from 17 kindergartens. A further study in China (2022) conducted an in-depth analysis of a single kindergarten using an extracellular vesicle (EV) fractionation approach, while another longitudinal study sampled three rooms in one kindergarten across five time points. Considerable methodological heterogeneity was observed among the included studies regarding ARG detection, bacterial quantification, and microbiome characterization. ARGs were identified using HT-qPCR combined with shotgun metagenomics (25.0%, n = 1), shotgun metagenomics alone (25.0%, n = 1), qPCR (25.0%, n = 1), and conventional PCR (25.0%, n = 1), while bacterial abundance was mainly quantified using 16S rRNA gene-based qPCR approaches (75.0%, n = 3). Microbiome characterization was primarily performed using 16S rRNA amplicon sequencing on Illumina HiSeq platforms, although one study used Roche 454 pyrosequencing and another applied shotgun metagenomic sequencing for higher-resolution taxonomic and functional profiling.

### 3.3. Distribution of ARG Classes and Resistance Genes in Kindergarten Dust Samples

**Figure 2:**
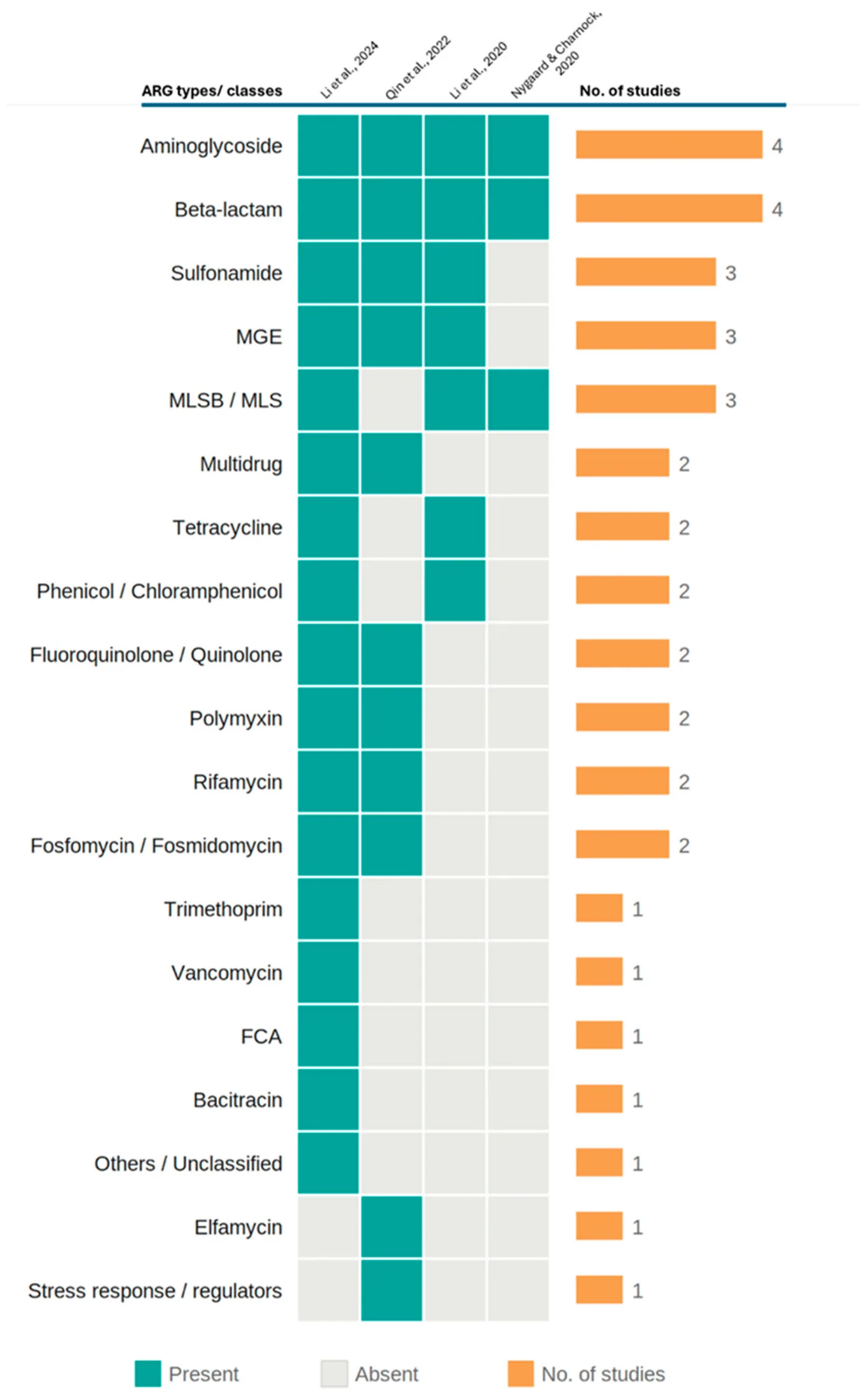
The ARG types/classes presented in four studies [15,16,17,18].

Across the included studies, three consistent findings emerged. ARGs were consistently detected in all kindergarten dust samples, regardless of country, season, sampling matrix, or detection method. Within the included studies, all sampled kindergarten dust matrices yielded at least one detectable ARG, although detection probability and apparent ARG richness varied substantially by analytical platform and target panel. No included study reported ARG-free samples; however, this observation is bounded by the four included studies and should not be generalized to all kindergartens. Sulfonamide, macrolide–lincosamide–streptogramin B (MLSB), tetracycline, beta-lactam, aminoglycoside, and multidrug resistance genes represent the consensus resistome shared across studies, were detected in at least two studies each and constitute the consensus resistome across the included studies (Figure 2). In addition, the most comprehensive studies consistently identified multidrug resistance genes as the predominant ARG class, accounting for the highest proportions in both outdoor dust metagenomes (50.9%) and EV-associated DNA (46.27%).

However, several important sources of heterogeneity should be considered when interpreting these findings. The reported richness of ARGs differed substantially according to the analytical approach used, ranging from only three ARG classes detected by PCR to 546 ARG subtypes identified through shotgun metagenomic sequencing. The methodological variation limits the validity of direct comparisons of overall ARG diversity across studies. These studies quantified ARG abundance using different normalization strategies, including copies per gram of dust, copies per 16S rRNA gene, copies per cell, and relative proportions of metagenomic reads. Because these abundance estimates were normalized using fundamentally different denominators, the reported ARG abundance metrics were not directly comparable and could not be meaningfully synthesized across studies. The seasonal variation remains poorly understood. Although one outdoor dust study conducted in Xiamen reported significantly greater ARG abundance during winter, none of the included indoor kindergarten dust studies evaluated seasonal effects. Consequently, the extent to which ARG exposure in kindergarten environments fluctuates across seasons remains unclear. The current evidence base is therefore sufficient to suggest the ubiquity and broad class diversity of ARGs in kindergarten dust, but insufficient to establish a reliable quantitative estimate of the ARG burden children are exposed to across different settings.

Differences in the reported dominant ARG classes should also be interpreted cautiously. Sulfonamide genes dominated by relative abundance in AC filter dust (Hong Kong), while multidrug resistance genes dominated in outdoor dust and EV-associated indoor dust (both China). This difference likely reflects the distinct sampling matrices rather than a true geographic difference, AC filter dust selectively accumulates airborne particulates over two-week periods, potentially concentrating specific ARG-carrying taxa, while floor and outdoor surface dust integrate a broader and more heterogeneous microbial input. This matrix effect is a critical confound that future studies should address by sampling multiple dust types within the same kindergarten simultaneously.

Multidrug resistance genes were the most extensively and consistently represented ARG class across studies, with numerous efflux pump-associated genes as shown in Table S4. Tetracycline resistance genes were notably diverse, encompassing ribosomal protection proteins, and efflux genes. Beta-lactam resistance genes were also detected across four studies. The greatest diversity was observed among aminoglycoside resistance genes, with multiple subtypes identified across two major enzymatic mechanisms: aminoglycoside acetyltransferases and aminoglycoside phosphotransferases. Multiple aminoglycoside-modifying enzyme genes, including acetyltransferase- and phosphotransferase-associated subtypes, were recurrently identified across studies. MLSB (macrolide–lincosamide–streptogramin B) resistance genes were reported in three of the four included studies. Notably, clinically significant ARGs such as *mecA* (methicillin resistance), *vanR*, and *vanY* (vancomycin resistance) were identified in kindergarten dust environments.

### 3.4. ARG and MGE Co-Occurrence

The detection of ARG-MGE co-occurrence across three of the four included studies using three different analytical approaches (network correlation analysis, metagenomic contig assembly, and targeted PCR) represents one of the strongest convergent findings in this review. The consistent identification of class 1 integron integrase (*intI1*) across indoor AC filter dust [16], indoor surface dust [18], and outdoor surface dust [17] is particularly significant, as *intI1* is the most clinically important MGE in terms of ARG mobilization and horizontal gene transfer in human-associated environments. Its detection in kindergarten dust across three distinct dust matrix indicates that the horizontal gene transfer machinery required for resistance spread is a stable and widespread feature of the kindergarten environment, not an isolated finding. The one study that did not assess MGE co-occurrence used endpoint PCR targeting only four ARG classes [15], the absence of MGE data from that study reflects a methodological limitation rather than a true biological absence.

### 3.5. Factors Associated with ARG Abundance

The included studies collectively identify season, urbanization, ventilation type, and occupancy as the principal modifiable environmental factors influencing ARG abundance and diversity in kindergarten dust, though the evidence for each varies substantially in strength and specificity. Seasonal variation and urbanization were significantly associated with higher ARG richness, abundance, and high-risk ARG counts in winter and in urban kindergartens (*p* < 0.05) across 118 samples collected from 59 kindergartens. These findings are internally consistent and replicated across both HT-qPCR and shotgun metagenomics analyses. In contrast, the evidence for ventilation type as a risk factor rests on just two studies using a single dust matrix (AC filter dust), both from the same city (Hong Kong and Xiamen, China), and neither designed to compare ventilated versus non-ventilated environments directly.

### 3.6. The Potential Exposure and Health Risk to Students

**Figure 3:**
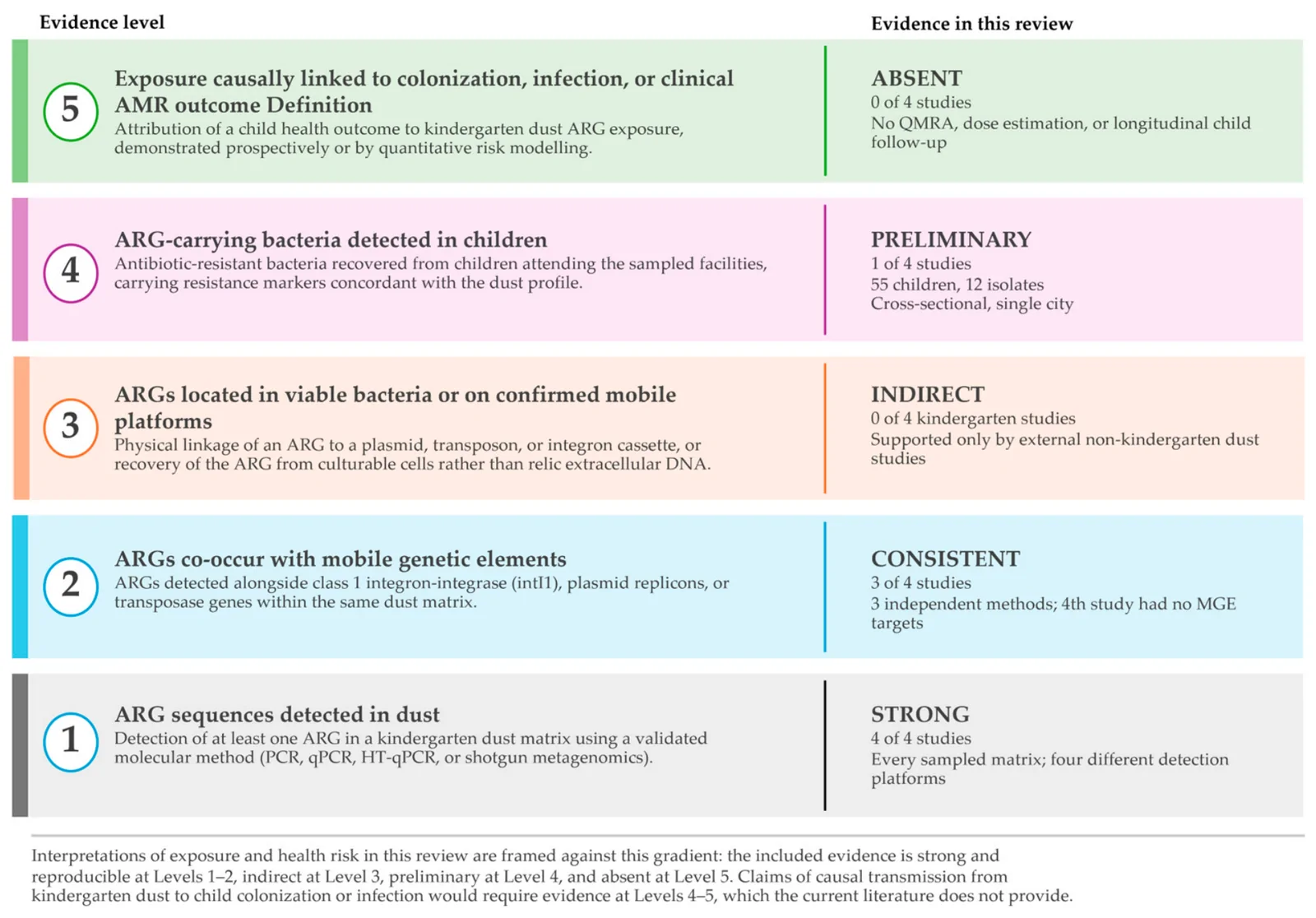
Evidence gradient along the putative kindergarten dust-to-child pathway. Levels 1–5 represent increasing inferential demand, from detection of ARG sequences in dust to demonstrated causal linkage with a clinical AMR outcome in children. Evidence from the four included studies is strong and reproducible at Levels 1–2, indirect at Level 3, preliminary at Level 4, and absent at Level 5.

To characterize the strength of evidence along the putative dust-to-child pathway, the findings were synthesized within a five-level evidence framework (Figure 3). Level 1 (detection of ARG sequences in dust) was supported by all four included studies, and Level 2 (co-occurrence of ARGs with mobile genetic elements [MGEs]) by three studies. Evidence for Level 3 (ARGs in viable bacteria or on confirmed mobile genetic platforms) remained indirect, as this was not directly shown in the included kindergarten studies. Level 4 (detection of ARG-carrying bacteria in children) was supported only by a single cross-sectional biomonitoring study, and no evidence was identified for Level 5, which requires a causal link to colonization, infection, or clinically relevant antimicrobial resistance outcomes. Overall, evidence was strong for environmental occurrence (Levels 1–2) but limited for biological transfer and absent for clinical outcomes (Levels 3–5). Accordingly, interpretations of potential health risk have been considered within this evidence gradient, recognizing that robust evidence currently supports environmental occurrence but not downstream biological or clinical effects.

The four included studies collectively provide an interconnected but still incomplete understanding of children’s ARG exposure in kindergarten environments. Three converging lines of evidence highlight a credible public health concern. First, ARGs were detected in every kindergarten dust matrix examined, including high-risk mobile Rank I ARGs and clinically important resistance determinants such as *mecA*, *intI1*, and *sul1*. Second, ARGs were consistently co-detected with potential human pathogens, including four of the six ESKAPE organisms (*Enterococcus faecium*, *Staphylococcus aureus*, *Klebsiella pneumoniae*, *Acinetobacter baumannii*, *Pseudomonas aeruginosa*, *Enterobacter* spp.) identified across three of the four studies. Third, the only direct human biomonitoring evidence identified ARB carrying *sul1*, *sul2*, *intI1*, *tetQ*, and *tetW* in urine samples from children attending the same kindergartens in which these genes predominated in AC filter dust profiles.

However, several important limitations qualify for these conclusions. No study in this review performed a formal quantitative health risk assessment, estimated inhalation doses, calculated disability-adjusted life years, or applied a probabilistic exposure model. The identification of potential pathogens across studies was based on sequence database matching rather than clinical culture or virulence confirmation, meaning that pathogenicity was inferred rather than experimentally confirmed. Furthermore, the single urine biomonitoring study (n = 55 children, 12 ARB isolates recovered) is too small and geographically restricted to support population-level conclusions about child ARB carriage attributable to kindergarten dust exposure. Collectively, the current evidence indicates that kindergarten dust environments may serve as reservoirs of ARGs, MGEs, and potential pathogens, whose co-occurrence could facilitate the acquisition or amplification of antibiotic resistance in young children. Establishing the actual magnitude of that risk requires prospective cohort studies with concurrent dust sampling and child microbiome or resistome monitoring over time.

### 3.7. Risk of Bias

The methodological quality of the four included studies was assessed using an adapted version of the Newcastle–Ottawa Scale (NOS) for observational studies, modified to reflect the cross-sectional and environmental exposure nature of kindergarten dust resistome research. A maximum of eight stars was therefore achievable. Studies scoring six or more stars were classified as low risk of bias; those scoring four to five stars as moderate risk; and those scoring three or fewer stars as high risk. The summary scoring of risk of bias assessment is presented in Table 3. Based on the narrative certainty appraisal, confidence in the synthesized findings was judged to be highest for the consistent detection of ARGs and for the co-occurrence of ARGs with class 1 integrons, both of which were consistent across multiple studies and detection platforms, and lowest for the seasonal, urbanization, and ventilation associations, each of which rested on a single study and is therefore regarded as preliminary. Because only four studies were included and no quantitative synthesis was undertaken, a formal statistical assessment of reporting bias due to missing results was not performed.

## 4. Discussion

### 4.1. Kindergarten Dust as a Ubiquitous and Persistent ARG Reservoir

The most distinguished finding of this systematic review is the consistent detection of antibiotic resistance genes (ARGs) across all kindergarten dust samples in all four included studies, regardless of country, season, indoor or outdoor location, or analytical method. No study identified ARG-free samples. This consistent observation across geographically and methodologically diverse studies conducted in China, Hong Kong, and Norway strongly supports the conclusion that ARGs are stable and persistent components of the kindergarten dust resistome rather than transient or opportunistic contaminants. This finding aligns with and extends previous evidence from other built environments. Similar to ARG detection in household dust [7], hospital indoor dust [10], and workplaces [29], our review establishes kindergartens as a category of the built environment that reliably harbors diverse ARGs. Collectively, these findings suggest that kindergarten dust represents a reservoir of antibiotic resistance genetic material, independent of country-specific antibiotic consumption patterns, climatic conditions, or building characteristics. Importantly, the exposed population in these settings consists of young children [15,24,25].

### 4.2. The Consensus Resistome: Interpreting the Dominant ARG Classes in Kindergarten Dust

The consensus resistome identified across geographically and methodologically diverse studies included sulfonamide, MLSB (macrolide–lincosamide–streptogramin B), tetracycline, beta-lactam, aminoglycoside, and multidrug resistance genes [15,16,17,18]. This cross-national convergence strongly suggests that these ARG classes are not incidental or environment-specific contaminants, but persistent components of the kindergarten dust resistome. This interpretation is further supported by broader indoor-environment resistome surveys from five diverse built environments, consistently recovered tetracycline (*tetW*), beta-lactam (*bla_SRT-1_*), MLSB (*ermB*), fluoroquinolone (*qnr*), and multidrug efflux genes as consensus indoor resistome constituents across settings [6]. Similarly, in a pilot study comparing resistomes from a pediatric hospital, maternity hospital, research offices, and households using HT-qPCR, identified MLSB and multidrug resistance genes as the highest-abundance classes across all building types [33]. Collectively, these findings position kindergartens as part of a continuum of human-occupied built environments that share a common consensus resistome are associated with human-associated microbial communities rather than setting-specific contamination sources. This interpretation is further supported by the two source-attribution analyses included in the reviewed studies, which suggested that β-lactam and macrolide resistance genes were associated with antibiotic consumption patterns in Hong Kong hospitals [16], while tetracycline and sulfonamide ARGs were attributed to cross-border agricultural sources from mainland China’s Pearl River Delta. In the Norwegian study, the three consistently detected ARGs (*mecA*, *ermA*, *aac(6′)-aph(2″)*) were primarily associated with *Staphylococcus* species [15], which are common human skin commensals. This finding supports the hypothesis that occupant shedding represents a major source of ARG contamination in kindergarten dust.

The origin of this consensus resistome is consistent with ARG profiles reported in human-occupied indoor environments. The dominance of multidrug resistance genes is mainly associated with efflux pump genes from the *mex*, *mdt*, and *emr* families. Efflux pumps represent a broadly adaptive resistance mechanism that confers simultaneous resistance to structurally diverse antibiotics and are commonly carried by human-associated bacteria [5]. Their predominance in kindergarten dust reinforces the interpretation that the primary source of ARGs in these environments is the human microbial reservoir (children and staff) rather than external environmental inputs such as agricultural runoff or industrial contamination.

Importantly, differences in the reported predominance of ARG classes across studies may be partially attributable to sampling matrix effects rather than true biological variation. AC-filter dust selectively concentrates airborne fine particles over accumulation periods of two weeks or more, potentially enriching specific ARG-carrying taxa such as the sulfonamide-resistant Proteobacteria [34], while floor and outdoor surface dust integrates a broader and more heterogeneous range of microbial inputs. This matrix effect is an important confound that has not yet been addressed within any single kindergarten study.

### 4.3. MGEs and the Horizontal Gene Transfer Dimension

Class 1 integrons, identified by the integrase gene *intI1*, are among the most important mobile genetic elements (MGEs) involved in the acquisition and dissemination of antibiotic resistance genes (ARGs) and are widely recognized as reliable indicators of anthropogenic resistome pollution because of their strong association with diverse ARG cassettes and horizontal gene transfer (HGT) [13,35]. Accordingly, the repeated detection of *intI1* in kindergarten dust across Hong Kong and China [16,17,18] suggests that resistance determinants in these environments are associated with mobile genetic platforms capable of facilitating gene exchange rather than existing solely as isolated genetic fragments. One of the most important findings of this review is the consistent co-occurrence of ARGs and MGEs, particularly *intI1*, in three of the four included studies [16,17,18]. The reproducibility of this observation across different geographic regions, dust matrices, and analytical platforms suggests that ARG-MGE linkage is a common feature of the kindergarten dust resistome rather than an isolated finding. This interpretation is further supported by evidence from an indoor dust metagenomic study demonstrating that 52 of 183 detected ARGs were physically located on plasmids, transposons, or integrons within viable bacteria [36], indicating that at least a proportion of indoor dust ARGs are associated with biologically relevant mobile platforms rather than extracellular relic DNA. Furthermore, the identification of broad-host-range conjugative plasmid replicons carrying clinically important ARGs in kindergarten dust reinforces the potential for resistance mobilization within indoor microbial communities [17].

Nevertheless, the current evidence supports the potential for ARG mobilization rather than active HGT. Although observational studies consistently demonstrate ARG-MGE co-occurrence and physical linkage, they do not establish whether gene transfer occurs in situ within kindergarten dust or quantify its frequency under environmental conditions. Addressing these questions will require experimental approaches, including conjugation or transformation assays and longitudinal microcosm studies capable of directly measuring gene transfer. Until such evidence becomes available, the available data indicate that kindergarten dust should be regarded as a plausible environmental reservoir of mobilizable resistance genes with the biological potential to contribute to antimicrobial resistance dissemination. Future environmental AMR surveillance would therefore benefit from incorporating MGE characterization, particularly *intI1* monitoring and plasmid replicon profiling, alongside conventional ARG detection.

### 4.4. Environmental and Seasonal Factors Associated with ARG Abundance

The included studies collectively suggest that season, urbanization, and ventilation type may influence ARG abundance and diversity in kindergarten dust. However, the evidence for each factor is limited in scope and should be interpreted with considerable caution, as each is supported by a small number of studies with important methodological constraints. Of the four included studies, only one study investigated seasonal effects on ARG profiles in outdoor kindergarten dust [17]. The winter excess in ARG burden with the number of ARG subtypes, absolute abundance, and Rank I ARG richness all significantly higher in winter than summer. Whether the winter ARG peak observed in Xiamen outdoor dust generalizes to indoor kindergarten environments, where temperature, humidity, and ventilation are partially regulated, or to kindergartens in non-subtropical climates, therefore remains entirely unknown from the current evidence base. Nevertheless, the directional finding of a winter ARG peak is biologically plausible and consistent with patterns reported in related environmental matrix outside the kindergarten context [37,38]. None of the included studies experimentally investigated the mechanisms underlying the higher winter ARG abundance. The only study reporting this seasonal pattern identified an association but did not establish causality. Therefore, the observed winter–summer difference should be regarded as a preliminary, hypothesis-generating finding requiring confirmation in longitudinal studies across diverse climatic regions.

Similarly, the association between urbanization and elevated ARG burden in kindergarten dust is supported by evidence from a single included study [17]. However, it derives from one city in China, and no other included study compared urban and non-urban kindergarten settings. The broader urbanization-resistome literature provides important contextual plausibility for this finding [39,40]. Nevertheless, the current evidence is insufficient to determine whether the urbanization effect generalizes beyond Chinese cities, applies equally to indoor and outdoor kindergarten dust, or holds across different climate zones and antibiotic use contexts.

The evidence concerning ventilation type are associated with ARG profiles in kindergarten dust is the most constrained of the three environmental factors considered in this review. Two included studies sampled AC-filter dust specifically from Hong Kong and Xiamen, China [16,17]. However, neither study was designed to compare mechanically ventilated versus naturally ventilated kindergarten environments directly, and both used AC-filter dust. The observed ARG profiles therefore describe what accumulates in AC-filter dust under mechanical ventilation conditions but cannot by themselves establish that mechanical ventilation increases kindergarten dust ARG burden relative to natural ventilation. Furthermore, because both studies were conducted in the same geographic region of subtropical coastal China, and used the same sample type, the possibility that their findings reflect regional microbial community characteristics rather than a ventilation-specific effect cannot be excluded.

Critically, the factors most readily targeted by school-level interventions, including cleaning frequency, disinfectant type, surface hygiene practices, and hand hygiene behavior, were either not measured or not systematically reported in any of the four included studies. The only direct evidence linking a specific building practice to microbial burden comes from a Norwegian study [15], which observed measurably better surface hygiene at a regularly cleaned countertop compared to an adjacent uncleaned ventilation hood, although ARG levels were not compared between these sites.

### 4.5. Evidence for Children’s Exposure and Health Risk

Beyond the diversity of ARG classes detected, the clinical significance of specific genes identified in kindergarten dust warrants particular attention. The detection of *mecA*, encoding methicillin resistance and conferring resistance to all beta-lactam antibiotics including cephalosporins and carbapenems in all floor dust samples throughout the 11-month longitudinal study in Norway [15], and in AC filter dust across all 17 Hong Kong kindergartens [16], points to the persistent presence of methicillin-resistant *Staphylococcus aureus* (MRSA)-type genetic material in kindergarten dust environments. The WHO has designated MRSA among the pathogens of highest priority for new antibiotic development given its limited treatment options and capacity for nosocomial and community-acquired infection [41]. Similarly, the detection of *vanR* and *vanY* (vancomycin resistance-associated genes) and *intI1* (class 1 integron integrase) carries substantial clinical implications.

Applying the ARG risk ranking framework [42], which classifies ARGs according to enrichment in human-associated environments, gene mobility, and host pathogenicity in ESKAPE organisms [17] identified 35 Rank I ARGs (current threats) and 11 Rank II ARGs (future threats) in outdoor kindergarten dust. The richness and abundance of Rank I ARGs were significantly higher in winter (*p* < 0.05), with winter mean abundance of 6.26 × 10^−3^ copies per cell compared to 3.18 × 10^−3^ copies per cell in summer. Notably, *lnuA*, a Rank I ARG conferring MLSB resistance, was detected in 100% of dust samples at an abundance of 3.4 × 10^−4^ copies per cell, representing the highest-abundance Rank I ARG detected. The prevalence of these high-risk ARGs in kindergarten outdoor dust at concentrations comparable to global wastewater treatment plant effluents [43], underscores the urgency of treating kindergarten dust as a legitimate AMR surveillance environment rather than a passive background reservoir.

The co-detection of ARGs with four of the six ESKAPE pathogen (*Acinetobacter baumannii*, *Enterococcus faecium*, *Pseudomonas aeruginosa*, *Staphylococcus aureus*) in three of the four included studies is a particularly significant finding from a clinical perspective [44]. ESKAPE pathogens have been designated Priority 1 organisms by the WHO, characterized by their multidrug resistance phenotypes, capacity for nosocomial infection, and limited treatment options [41]. In kindergarten dust, ESKAPE-associated organisms were identified by metagenomic sequence classification only [17] and metagenomic contig assembly [18]; pathogenicity and viability were inferred from sequence homology and were not confirmed by culture or virulence assays. Culture-based isolation was applied only to antibiotic-resistant bacteria recovered from children’s urine [16]. The co-occurrence of ESKAPE pathogens with Class 1 integrons was confirmed through network co-occurrence analysis [17], contig assembly [18], and urine biomonitoring [16]. Class 1 integrons are the single most important mobile platform for ARG capture, dissemination, and expression in clinical pathogens, with their hosts predominantly found within the ESKAPE pathogen group [35,44]. This finding suggests that kindergarten dust may not only expose children to resistant genes but to resistant pathogens carrying those genes on transferable genetic elements, thereby substantially increasing the potential clinical risk beyond the presence of ARGs alone.

The most direct and compelling evidence of health risk to children from dust-associated ARGs comes from the urine biomonitoring component [16], which identified antibiotic-resistant bacteria (ARB) in urine samples from 55 Hong Kong kindergarten children. Among the 12 ARB strains recovered, 10 were multidrug resistant and 10 carried the integron gene *intI1*, while *sul1* was detected in all urinary ARB isolates. The concordance between the dominant ARG profiles detected in AC filter dust (*sul1*, *sul2*, and *intI1*) and those identified in urinary ARB isolates from children attending the same kindergartens represents the strongest epidemiological signal identified in this review. Although the cross-sectional design precludes causal inference, and alternative acquisition routes such as food, household environments, or prior antibiotic exposure cannot be excluded, the temporal and spatial overlap between environmental ARG profiles and child urinary ARB profiles is consistent with the proposed dust-to-child exposure pathway. This pathway is biologically plausible because concentrations of resuspended floor-dust bacteria are substantially higher in the infant breathing zone than in the adult breathing zone [24,25], while frequent hand-to-mouth behaviors provide additional opportunities for ingestion of dust-associated microorganisms and ARGs [26,27].

The detection of these last-resort resistance determinants in a child-dense, non-clinical indoor environment warrants serious attention. Global AMR burden analyses estimate that antibiotic-resistant infections caused approximately 1.27 million direct deaths in 2019 [1] and 1.14 million direct deaths in 2021 [2], with ESKAPE pathogens carrying carbapenem and vancomycin resistance genes disproportionately contributing to fatal outcomes. Although the detection of *bla_NDM_*, *vanA*, and *mcr-5* genes in kindergarten dust does not in itself confirm transmission and infection risk, their presence in an environment attended daily by young children represents a pre-cautionary concern that justifies formal exposure assessment [26,27].

### 4.6. Methodological Heterogeneity and Its Implications for Evidence Synthesis

A major constraint on the conclusions that can be drawn from this review is the substantial methodological heterogeneity across the four included studies, which precluded quantitative pooling of any outcomes. Differences in ARG detection platforms, abundance normalization strategies, sampling matrices, and sample sizes limit the degree to which findings can be meaningfully compared or synthesized across studies. Understanding the nature and implications of this heterogeneity is essential not only for contextualizing the current evidence base but also for guiding the design of future kindergarten dust resistome studies toward greater comparability.

The four included studies employed four different ARG detection platforms including conventional endpoint PCR [15], targeted qPCR [16], HT-qPCR combined with shotgun metagenomics [17], and shotgun metagenomics [18], none of which were identical. The included studies used a range of molecular detection methods with differing analytical strengths and limitations. Conventional PCR provides qualitative detection of selected targets with limited sensitivity, while targeted qPCR enables quantitative measurement of predefined genes. HT-qPCR expands this approach to hundreds of ARGs and MGEs simultaneously but remains restricted to known targets included in the array design. In contrast, shotgun metagenomics offers broader, sequence-independent detection of all DNA within a sample, although sensitivity depends on sequencing depth, and results are typically reported as relative rather than absolute abundances. This methodological diversity had profound consequences for the apparent richness and diversity of ARGs detected across studies. This more than 100-fold difference in detected ARG diversity should not be interpreted as a biological difference in the resistance gene burden of Norwegian versus Chinese kindergartens. These discrepancies are therefore more plausibly explained by differences in the sensitivity and analytical coverage of the detection platforms rather than by true biological variation. These methodological limitations are consistent with previous comparative studies showing that HT-qPCR and shotgun metagenomics capture largely non-overlapping portions of the indoor dust resistome, reflecting substantial differences in detection sensitivity and analytical coverage [33]. Similarly, the comparing qPCR and metagenomic sequencing in wastewater resistome profiling reported imperfect concordance across all antibiotic resistance classes examined [45]. These findings indicate that ARG richness estimates are strongly influenced by the detection platform used, meaning that cross-study comparisons of ARG diversity and abundance must account for methodological differences. Accordingly, future meta-analyses should either include studies using comparable analytical platforms or apply platform-specific correction approaches.

Furthermore, the difference in sample collection methods and normalizing of ARG abundance should be carefully considered when interpreting cross-study comparisons and synthesizing findings across studies. The included studies used different dust matrix and sampling approaches, including AC-filter dust, settled floor dust, pooled indoor dust, and outdoor surface dust collected by either vacuum sampling or passive accumulation. These matrixes differ in their sources, particle accumulation characteristics, and microbial composition, which independently influence ARG profiles. Consequently, ARG findings across studies are not directly comparable, even when similar analytical platforms are used. In addition, pooled sampling strategies may obscure important spatial variation within kindergarten environments. These findings highlight the need for standardized sampling strategies, ideally involving simultaneous collection of multiple dust matrix from the same facility, to improve comparability across future studies. Also, included studies used fundamentally different approaches for quantifying and normalizing ARG abundance, including copies per gram of dust, normalization to 16S rRNA gene copies, relative metagenomic read abundance, and qualitative presence–absence reporting. Because these methods rely on different denominators and analytical assumptions, ARG abundance data across studies are not directly comparable and cannot be quantitatively pooled. Standardization of normalization approaches therefore remains a major methodological priority for future indoor dust resistome research. Importantly, these heterogeneities reflect the early and rapidly evolving state of the field: no standardized protocol yet exists for the collection, processing, or analysis of dust resistomes in school environments.

### 4.7. Limitations of This Review

Several limitations of this systematic review should be acknowledged. First, only four studies met all inclusion criteria, reflecting the genuine scarcity of evidence on ARGs specifically in kindergarten dust and limiting the generalizability of conclusions. Also, this review focused exclusively on kindergartens, excluding primary schools, secondary schools, and universities, where ARG exposure patterns may differ. However, the small study base reflects the genuinely nascent state of research on ARGs in early childhood educational environments rather than a deficiency in the search strategy, but it means that the conclusions of this review are best understood as a mapping of current knowledge and an identification of evidence gaps rather than a definitive synthesis of an established literature. Second, the limited geographic coverage of the included studies that restricted to China (including Hong Kong) and Norway, may limit the generalizability of the findings to other global settings, especially low- and middle-income countries. The current evidence base is geographically limited, making it unclear whether the ARG profiles, MGE distributions, and environmental factors observed in this review are representative of kindergarten environments in other global regions, particularly those with high burdens of pediatric AMR [2]. This geographic imbalance substantially limits the global generalizability and policy relevance of the findings. Third, the substantial methodological heterogeneity across studies, including differences in ARG detection platforms, abundance normalization strategies, and dust sampling matrices. These inconsistencies prevented quantitative meta-analysis and limited comparisons to qualitative interpretation, as reported ARG diversity and abundance were strongly influenced by methodological differences rather than biological variation alone. Fourth, all included studies used observational designs, limiting the ability to establish causal relationships between kindergarten dust ARG exposure and child health outcomes. None of the studies performed formal quantitative risk assessments, exposure-dose estimation, or probabilistic exposure modelling, while pathogen identification was largely based on sequence matching rather than direct clinical or virulence confirmation. Although one biomonitoring study detected antibiotic-resistant bacteria in children’s urine, the cross-sectional design could not determine whether these organisms originated from kindergarten dust exposure or other environmental sources.

### 4.8. Implications for Policy, Practice, and Future Research

Although the current evidence base is insufficient to support definitive quantitative exposure limits or mandatory regulatory standards for ARGs in kindergarten dust, it is sufficient to justify several precautionary policy considerations. The findings of this review carry several actionable implications for public health policy and practice. At the institutional level, the consistent detection of clinically significant ARGs including *mecA*, *intI1*, *sul1*, and ESKAPE pathogen-associated genes in kindergarten dust across multiple countries argues for the establishment of ARG monitoring as a routine component of kindergarten indoor environmental quality assessment. In addition, no international or national standard defines acceptable ARG levels in kindergarten indoor environments. Therefore, kindergartens should be considered a priority setting for future environmental AMR surveillance research; whether they should ultimately be incorporated into national or international surveillance frameworks will depend on standardized, multi-site data that do not yet exist. Building operators and public health authorities should recognize that AC-filter dust in mechanically ventilated kindergartens may accumulate clinically relevant ARGs. This observation raises the hypothesis that filter management and ventilation practices could influence ARG accumulation in indoor dust. However, this hypothesis requires validation through well-designed intervention studies before evidence-based recommendations can be made.

Future research should prioritize methodological standardization, including harmonized ARG detection, normalization, and sampling protocols, to enable reliable cross-study comparison and meta-analysis. Simultaneous multi-matrix sampling (AC-filter, floor, and outdoor dust) and longitudinal multi-season studies are needed to clarify the effects of seasonality, urbanization, and ventilation on kindergarten dust resistomes. Intervention-based studies evaluating ventilation systems, filter replacement frequency, and cleaning practices should be conducted to identify effective strategies for reducing ARG accumulation indoors. Finally, formal quantitative microbial risk assessment (QMRA) and prospective child cohort studies integrating environmental and child resistome monitoring are critically needed to determine the actual health risks associated with kindergarten dust ARG exposure.

## 5. Conclusions

Across four studies conducted in China, Hong Kong, and Norway between 2018 and 2024, ARGs were consistently detected in every kindergarten dust matrix examined. The consensus resistome comprising sulfonamide, macrolide–lincosamide–streptogramin B (MLSB), tetracycline, β-lactam, aminoglycoside, and multidrug resistance genes was reproducibly identified across studies. Three of the four studies further reported the co-occurrence of ARGs with mobile genetic elements, particularly class 1 integrons (*intI1*), indicating that resistance determinants are associated with genetic platforms capable of facilitating horizontal gene transfer, although active transfer has not yet been demonstrated. Together, these Level 1–2 findings were reproducible across geographic regions, dust matrices, and analytical platforms, and constitute the strongest evidence synthesized in this review.

In contrast, evidence regarding the magnitude of children’s exposure, the viability of ARG-carrying microorganisms, the frequency of in situ horizontal gene transfer, the environmental sources of specific ARG classes, and the influence of seasonality, urbanization, and ventilation remains limited to individual studies or indirect inference and should be regarded as hypothesis-generating rather than confirmatory. Although a single cross-sectional urine biomonitoring study is compatible with a potential dust-to-child exposure pathway, it cannot establish causality, and no study has demonstrated that kindergarten dust-derived ARGs contribute to microbial colonization, infection, or clinically relevant antimicrobial resistance outcomes (Levels 4–5).

Future research should prioritize standardized protocols for dust sampling, DNA extraction, sequencing, and data normalization to improve cross-study comparability. Well-powered longitudinal investigations spanning multiple geographic regions, climatic zones, and socioeconomic settings are needed, together with child-linked prospective cohort studies integrating environmental and human resistome surveillance. Experimental studies, including conjugation and transformation assays, should directly quantify the frequency of horizontal gene transfer within dust microbial communities, and intervention trials evaluating ventilation, air filtration, and cleaning strategies should assess their effectiveness in reducing ARG abundance. Finally, quantitative microbial risk assessment (QMRA) frameworks are needed to translate environmental ARG occurrence into estimates of human health risk. Until such evidence becomes available, kindergarten dust should be regarded as a biologically plausible—but not yet established—environmental exposure pathway for antimicrobial resistance that warrants precautionary attention within a One Health framework.

## Figures and Tables

**Table 1:** Inclusion and exclusion criteria applied in the systematic literature search.

Inclusion Criteria	Exclusion Criteria
1. Studies examining dust collected from kindergartens or preschool educational or daycare environments attended by children aged 1–6 years	1. Studies conducted in non-kindergarten school settings (primary schools, secondary schools, universities) without a specific kindergarten/preschool/daycare component
2. Studies reporting at least one quantitative or qualitative antibiotic resistance gene (ARG) outcome in dust samples	2. Studies examining air samples, surface swabs, or soil samples not characterized as settled or collected dust
3. Studies using any validated molecular method for ARG detection (PCR, qPCR, High-throughput qPCR, shotgun metagenomics)	3. Studies reporting microbial community profiling only, without ARG-specific outcomes
4. Studies published in English	4. Reviews, meta-analyses, editorials, conference abstracts, and grey literature
5. Primary research articles (original studies)	5. Studies not published in English
6. Both indoor and outdoor dust sampling matrices within kindergarten premises	6. Studies for which full-text access could not be obtained

**Table 2:** Summarizes the characteristics of studies on antibiotic resistance genes (ARGs) in dust from kindergarten.

Author & Year	Country	Design	Sample Site/Type	Sampling Period	Samples (n)	Sampling Methods	Args Detection	Targeted ARGs	16S rRNA Quantified	Key Comparability Limitation	References
Li et al. (2024)	China	Cross-sectional	Outdoor (windowsills, railings, roller shutter doors)	Winter (January 2020) Summer (June 2020)	118 (59 winter + 59 summer)	Vacuum collection	HT-qPCR and Shotgun metagenomics	HT-qPCR panel: 319 ARG subtypes, 57 MGE genes (34 transposase, 11 plasmid, 9 insertion sequence, 3 integron-integrase); whole-metagenome sequencing (CARD database)	HT-qPCR	Outdoor dust only; results may not represent the indoor breathing zone	[17]
Qin et al. (2022)	China	Cross-sectional	Indoor (floor, shelves, table, AC filters)	Summer (October 2020)	1 pooled sample	Vacuum collection	Shotgun metagenomics	Whole-metagenome sequencing (CARD database); 241 ARG subtypes across 16 resistance classes in EV fraction	SYBR Green qPCR	Single pooled sample from one kindergarten; EV fraction only; no replication	[18]
Li et al. (2020)	China (HK)	Cross-sectional	Indoor (AC filter dust, classrooms)	Summer (June–October 2017)	17 AC filter dust samples	Passive dust collection	qPCR	6 ARG classes; 22 ARG subtypes; 1 MGE gene (*intI1*)	SYBR Green qPCR	AC-filter dust only; targeted qPCR panel; limited (12-isolate) child biomonitoring	[16]
Nygaard et al. (2018)	Norway	Longitudinal	Indoor (floor dust, 3 rooms)	February 2015–January 2016 (11 months)	15 floor dust samples	Vacuum collection	Conventional PCR	4 ARG classes; 4 ARG subtypes (*mecA*, *ermA*, *aac(6′)-aph(2″)*, *vanA*)	Not examined	Qualitative (presence/absence) only; no MGE data; no 16S quantification	[15]

Noted: For [15] study, ARG data are qualitative (presence/absence) only.

**Table 3:** Assessment of bias of the included studies using the Newcastle–Ottawa Scale (NOS).

	Risk of Bias Domains								
Study	D1	D2	D3	D4	D5	D6	D7	D8	Quality
Nygaard & Charnock, 2018 [15]	★	★	☆	☆	☆	★	☆	☆	High risk
Li et al., 2020 [16]	★	★	★	★	☆	★	★	☆	Low risk
Li et al., 2024 [17]	★	★	★	★	★	★	★	☆	Low risk
Qin et al., 2022 [18]	☆	★	★	★	☆	★	★	☆	Moderate risk

Domains: D1: Representativeness of the study sample; D2: Ascertainment of the exposure (dust sampling); D3: Presence of a comparator; D4: Control for the main confounder (bacterial biomass); D5: Control for additional confounders (season, setting, methodology); D6: Validity of the ARG detection method; D7: Adequacy of ARG quantification; D8: Adequacy of follow-up/exposure-outcome assessment. Scoring: ★ = criterion met; ☆ = criterion not met; Low risk ≥ 6 ★; Moderate risk 4–5 ★; High risk ≤ 3 ★.

## Data Availability

The data presented in this study are available from the corresponding author upon reasonable request.

## Supplementary Materials

The following supporting information can be downloaded at: https://www.mdpi.com/article/10.3390/ijerph23081036/s1, Table S1: Database search strings used for the systematic literature search (conducted March 2026); Table S2: Rationale for the adaptation of the Newcastle–Ottawa Scale (NOS) to environmental kindergarten dust resistome studies, item-level justification of scoring, and target-specific risk-of-bias ratings; Table S3: Item-level justification of scoring, and target-specific risk-of-bias ratings; Table S4. Distribution of antibiotic resistance gene (ARG) classes across the four included studies, classified as universally reported (all four studies), consensus (≥2 studies), or study-specific expanded resistome (single high-resolution study); Supplementary Materials File: PRISMA checklist.
