# Supplementary material for: Antibiotic Resistance Genes in Dust from Kindergarten Environments: A Systematic Review of Occurrence, Diversity, Determinants, and Exposure Implications
Source: Int J Environ Res Public Health. 2026 Aug 9;23(8):1036. doi: 10.3390/ijerph23081036 (PMC13513710; doi:10.3390/ijerph23081036)
Supplement: Supplementary file 1 [file ijerph-23-01036-s001.zip › Supplementary File.pdf]

Table S1. Database search strings used for the systematic literature search (conducted March 2026).

Database	Search Date	Search String
**PubMed**	March 2026	("Drug Resistance, Bacterial"[Mesh] OR "Antimicrobial Resistance"[Title/Abstract] OR "Antibiotic Resistance"[Title/Abstract] OR "drug–resistant bacteria"[Title/Abstract] OR "antibiotic resistant bacteria"[Title/Abstract] OR "resistance gene*"[Title/Abstract] OR "antibiotic resistance gene*"[Title/Abstract] OR ARG[Title/Abstract] OR ARGs[Title/Abstract] OR resistome[Title/Abstract])<br>AND<br>("Dust"[Mesh] OR dust[Title/Abstract] OR "indoor dust"[Title/Abstract] OR "settled dust"[Title/Abstract] OR "floor dust"[Title/Abstract] OR "surface dust"[Title/Abstract] OR "house dust"[Title/Abstract] OR "environmental dust"[Title/Abstract])<br>AND<br>("Schools"[Mesh] OR school*[Title/Abstract] OR classroom*[Title/Abstract] OR "educational environment*"[Title/Abstract] OR "educational building*"[Title/Abstract] OR daycare[Title/Abstract] OR "day care"[Title/Abstract] OR kindergarten*[Title/Abstract] OR preschool*[Title/Abstract] OR "child care center*"[Title/Abstract] OR dormitor*[Title/Abstract] OR universit*[Title/Abstract])
**Scopus**	March 2026	(TITLE–ABS–KEY("antibiotic resistance" OR "antimicrobial resistance" OR "drug resistant bacteria" OR "antibiotic resistant bacteria" OR "resistance gene*" OR "antibiotic resistance gene*" OR ARG OR ARGs OR resistome))<br>AND<br>(TITLE–ABS–KEY(dust OR "indoor dust" OR "settled dust" OR "floor dust" OR "surface dust" OR "environmental dust"))<br>AND<br>(TITLE–ABS–KEY(school* OR classroom* OR "educational environment*" OR "educational building*" OR daycare OR "day care" OR kindergarten* OR preschool* OR "child care center*" OR dormitor* OR universit*))
**Web of Science**	March 2026	TS=(("antibiotic resistance" OR "antimicrobial resistance" OR "drug resistant bacteria" OR "antibiotic resistant bacteria" OR "resistance gene*" OR "antibiotic resistance gene*" OR ARG OR ARGs OR resistome)<br>AND

	(dust OR "indoor dust" OR "settled dust" OR "floor dust" OR "surface dust" OR "environmental dust")<br>AND<br>(school* OR classroom* OR "educational environment*" OR "educational building*" OR daycare OR "day care" OR kindergarten* OR preschool* OR "child care center*" OR dormitor* OR universit*))

Table S2. Rationale for the adaptation of the Newcastle–Ottawa Scale (NOS) to environmental kindergarten dust resistome studies, item-level justification of scoring, and target-specific risk-of-bias ratings.

ID	Adapted domain	Original NOS item (domain)	Rationale for adaptation	Criterion for awarding ★	Inferential target informed
**D1**	**Representativeness of the study sample**	Item 1 — Representativeness of the exposed cohort (Selection)	In environmental resistome studies the "cohort" is the set of sampled facilities and sampling points rather than a group of persons. The item was retained but re-specified so that representativeness refers to whether kindergartens and sampling locations were drawn from a defined frame rather than selected opportunistically.	Multiple kindergartens sampled from a defined sampling frame, OR all eligible sampling locations within a single facility systematically enumerated and sampled. ☆ where a single convenience or pooled sample was taken without a defined frame.	ARG detection
**D2**	**Ascertainment of the exposure (dust sampling)**	Item 3 — Ascertainment of exposure (Selection)	Retained with minimal change; the "exposure" is redefined as the dust matrix itself. A reproducibly described sampling procedure is the environmental analogue of secure exposure records.	Sampling matrix, collection device, surface area or mass collected, and storage/transport conditions reported in sufficient detail to permit replication. ☆ where the method is named without operational detail.	ARG detection; abundance
**D3**	**Presence of a comparator**	Item 2 — Selection of the non-exposed cohort (Selection)	An unexposed cohort is not meaningful where every sample is by definition exposed. The item was re-specified as the presence of any internal contrast (seasonal, spatial, urban/suburban, matrix-type, or fraction-based) against which ARG profiles can be interpreted.	At least one prespecified internal comparison group or stratum reported. ☆ where a single undifferentiated sample set was analyzed.	Determinants (descriptive only)

			This domain legitimately down-weights purely descriptive designs for causal purposes, but does not bear on whether ARG detection itself was reliable.		
**D4**	**Control for the main confounder (bacterial biomass)**	Item 5a — Comparability, most important factor (Comparability)	The dominant confounder of apparent ARG abundance in dust is total bacterial load: an apparent increase in ARGs may reflect more bacteria rather than more resistance. Quantification of 16S rRNA gene copies is therefore the environmental analogue of adjusting for the principal confounder.	Bacterial biomass quantified (16S rRNA gene qPCR or equivalent) and ARG results normalized or interpreted with reference to it. ☆ where biomass was not measured.	ARG abundance
**D5**	**Control for additional confounders (season, setting, methodology)**	Item 5b — Comparability, additional factor (Comparability)	Retained, with the additional factors specified a priori as those most likely to distort ARG comparisons in this literature: sampling season, urban versus suburban setting, and analytical platform.	At least one additional confounder measured and addressed in the design or analysis. ☆ otherwise.	Determinants (descriptive only)
**D6**	**Validity of the ARG detection method**	Item 6 — Assessment of outcome (Outcome)	The "outcome" is ARG detection. Blinded independent outcome assessment is not applicable to molecular assays, so the criterion was re-specified in terms of assay validity and quality control.	Validated molecular platform with reported primers, panel, or annotation database, together with negative and positive controls or equivalent quality control. ☆ otherwise.	ARG detection
**D7**	**Adequacy of ARG quantification**	Item 7 — Was follow-up long enough for outcomes to occur (Outcome)	Duration of follow-up is meaningless for a cross-sectional environmental measurement. The item was replaced with the closest functional equivalent for this literature: whether the outcome was	ARGs quantified on an absolute or relative scale with a stated normalization denominator. ☆ where reporting was presence/absence only.	ARG abundance

			measured on a quantitative scale at all, since presence/absence data cannot support abundance comparison or pooling.		
**D8**	**Adequacy of follow-up / exposure–outcome assessment**	Item 8 — Adequacy of follow-up of cohorts (Outcome)	Re-specified as whether the study linked measured dust ARG exposure to any downstream biological or health outcome in the exposed children. This domain was retained deliberately, despite penalizing descriptive studies, because it makes transparent why none of the included studies can support causal or health-risk inference.	Concurrent or prospective assessment of an ARG-related outcome in the exposed children, with reported participation or completeness. ☆ otherwise.	Child health risk (descriptive only)

Item omitted. NOS Selection item 4 ("demonstration that the outcome of interest was not present at the start of the study") was removed as inapplicable: ARGs are ubiquitous background constituents of indoor dust and a resistome-free baseline cannot be established. The maximum achievable score is therefore eight stars rather than nine.

Composite thresholds. Low risk ≥ 6 ★; moderate risk 4–5 ★; high risk ≤ 3 ★, as reported in Table 3 of the main text.

Table S3. Item-level justification of scoring, and target-specific risk-of-bias ratings.

Domain	Nygaard & Charnock, 2018 [15] — Norway	Li et al., 2020 [16] — Hong Kong SAR	Li et al., 2024 [17] — China	Qin et al., 2022 [18] — China
**D1 Representativeness**	★ All three occupied rooms of the facility systematically sampled at five time points over 11 months; the within-facility sampling frame was fully enumerated.	★ Seventeen kindergartens sampled across districts, giving a multi-facility rather than a convenience sample.	★ Fifty-nine kindergartens sampled across urban and suburban strata; the largest and most systematically framed sample in this review.	☆ A single pooled sample from one kindergarten; no sampling frame, and pooling precludes assessment of within- or between-facility variation.
**D2 Exposure ascertainment**	★ Vacuum collection of floor dust, with matrix, rooms, and repeated sampling schedule reported.	★ Passive AC-filter dust accumulation, with filter type, accumulation period, and handling described.	★ Vacuum collection from specified outdoor surfaces, replicated across two seasons.	★ Vacuum collection with an extracellular vesicle fractionation protocol described in replicable detail.
**D3 Comparator**	☆ No internal comparator; a single facility with no seasonal, spatial, or matrix contrast analyzed for ARGs.	★ Between-kindergarten and district-level contrasts reported; dust and urinary ARB profiles compared.	★ Prespecified winter versus summer and urban versus suburban contrasts.	★ EV-associated versus non-EV (whole-dust) DNA fractions compared as an internal contrast.
**D4 Main confounder (biomass)**	☆ 16S rRNA gene copies not quantified; ARG results cannot be referenced to bacterial biomass.	★ 16S rRNA gene quantified by SYBR Green qPCR and used for normalization.	★ 16S rRNA quantified within the HT-qPCR array and used for normalization (copies per cell).	★ 16S rRNA gene quantified by SYBR Green qPCR.
**D5 Additional confounders**	☆ Season, setting, and analytical platform neither varied nor adjusted for.	☆ Single season (June–October 2017); no seasonal or platform contrast.	★ Season and setting measured and analyzed; two platforms (HT-qPCR and shotgun metagenomics) cross-compared.	☆ Single season, single site, single platform; no additional confounder addressed.

**D6 Detection method validity**	★ Conventional endpoint PCR using defined published primers for four targets.	★ Targeted qPCR with published assays for 22 ARG subtypes and intI1.	★ Validated HT-qPCR panel (319 ARG subtypes, 57 MGEs) plus CARD-annotated shotgun metagenomics.	★ Shotgun metagenomics with CARD annotation.
**D7 Quantification adequacy**	☆ Qualitative presence/absence reporting only; no quantitative scale or normalization denominator.	★ Absolute quantification with a stated normalization denominator.	★ Absolute and relative quantification with explicit denominators.	★ Relative abundance quantified with a stated denominator (proportion of metagenomic reads).
**D8 Exposure–outcome assessment**	☆ No child biological or health outcome assessed.	☆ Urinary ARB sampling was concurrent and cross-sectional, with no follow-up and no linkage of individual children to their own classroom dust.	☆ No child biological or health outcome assessed.	☆ No child biological or health outcome assessed.
**Composite score**	**3 / 8 — High risk**	**6 / 8 — Low risk**	**7 / 8 — Low risk**	**5 / 8 — Moderate risk**

★ = criterion met; ☆ = criterion not met. Scores correspond exactly to those reported in Table 3 of the main text.

Table S4. Distribution of antibiotic resistance gene (ARG) classes across the four included studies, classified as universally reported (all four studies), consensus (≥2 studies), or study-specific expanded resistome (single high-resolution study).

No.	ARG class	Mechanism	L. J. Li et al., 2024		Qin et al., 2022		N. Li et al., 2020		Nygaard & Charnock, 2018	
			n	*Gene subtypes*	*n*	*Gene subtypes*	*n*	*Gene subtypes*	*n*	*Gene subtypes*
1	**Aminoglycoside**	Enzymatic modification	52	*aacC2*, *aacA–aphD*, *AAC(6')–Iia*, *aphA3*, *aac(6')–Ib*, *aadA5/6/7/10/16/17/21*, *aadA*, *aadA_99*, *aadB*, *aadD*, *aadE*, *aac(3)–I/II/III/IV/IIIa/ib/Ia/Via*, *aac(6')–I/II/ig/im/ir/iv/iw/Ie–APH(2'')–Ia–2*, *APH(3')–VIa*, *aph4ib*, *aph6ic*, *aph(3')–I*, *aph(3'')–I/ia*, *aph(4)–Ia*, *aph(6)–I*, *aph(3''')–III*, *aph3–III*, *APH(6)–Ia*, *ant(6)–Ia*, *ant(9)–I*, *apmA*, *aacC4*, *aac(2')–I*, *spcN*, *bifunctional AAC–APH*, *streptomycin resistance protein*	1	*APH(6)–Id*	1	*aac(6')–Ib*	1	*aac(6')–aph(2'')*
2	**Beta–lactam**	Enzymatic inactivation / Target modification	26	*blaTEM*, *blaSHV–11*, *blaVEB*, *blaROB*, *blaGES*, *blaOXA10*, *blaOXY–1*, *blaCARB*, *blaHERA*, *blaADC*, *blaBEL*, *bla–ACT*, *bla–L1*, *blaMOX/blaCMY*, *PSE–1*, *NDM*, *class A/C beta–lactamase*, *metallo–beta–lactamase*, *beta_ccra*, *cefa_ampc*, *penA*, *PBP–1A*, *PBP–2X*, *OXA–12*, *THIN–B*	3	*MOX–5/MOX–7*, *PDC–5*, *OXA–50*	1	*mecA*	1	*mecA*
3	**Tetracycline**	Efflux / Ribosomal protection	36	*tetA/B/C/D/E/G/L/M/O/P/Q/R/S/T/V/W/Z*, *tetX2*, *tet32/35/36/39/40/43/44*, *tetB(P)*, *tetA(P)*, *otrA*, *tetracycline resistance protein*	–	–	5	*tetW*, *tetS*, *tetM*, *tetQ*, *tetO*	–	–
4	**Multidrug**	Efflux pump overexpression	71	*mexA/B/C/D/E/F/I/T/W/Y*, *oprC/D/J/M/N*, *mdtA/B/C/D/E/F/G/H/K/L/M/N/P*, *acrA/B/F*, *adeB/C/J/K*, *emrA/B/D/E*, *smeB/D/E*, *ceoB*, *cmeB*, *bpeF*, *amrB*, *mdfA*, *norA*, *mtrD*, *TolC/tolC*, *cfr*, *msr(E)*, *optrA*, *vgaA*, *sugE*, *mepA*, *qacEdelta1*, *qacH_351*, *EmrB–QacA MFS transporter*, *multidrug ABC transporter*, *oqxA*, *mefA*	13	*mexK*, *mexW*, *mexF*, *mexI*, *mexD*, *mexX*, *mexH*, *mexM*, *mdtK*, *emrB/emrD*, *mdtB/mdtC/mdtH*, *mexA/mexC/mexE*, *TriC*	–	–	–	–
5	**MLSB**	rRNA methylation / Efflux / Enzymatic inactivation	35	*ermA/B/E/F/O/Q/S/X*, *erm(35/36/39/K)*, *ermA_ermTR*, *lnuA/B/C/F*, *vat(A/E)*,	–	–	4	*ermA*, *ermB*, *ermC*, *ermF*	1	*ermA*

				ere(A/B), ereA, mphA/C, lmrA, oleC, macA/B, mefA, lsa						
6	**Mobile Genetic Element (MGE)**	Horizontal gene transfer	35	*intI1/intI3, int1, IS26/256/91/613/630/6100, IS1133/1247, IS200–1/2, IS6/257, IS21–ISAs29, ISAba3, ISCR1, ISEcp1, ISEfm1, ISPps1, ISSm2, Tn3, TN5403, tnpA–5/6/7, mobA, traN, trb–C, trfa, Tp614, orf37/39–IS26, IncN_rep, IncP_oriT, pBS228–IncP–1α*	6	*intI, intI1, intI2, intI4, intI9, intIq*	2	*intI1, int1*	–	–
7	**Sulfonamide**	Target bypass (DHPS inhibition)	5	*sul1, sul2, sul4, sulA_folP*	1	*folP*	2	*sul1, sul2*	–	–
8	**Trimethoprim**	Target bypass (DHFR inhibition)	10	*dfrA1, dfrA10, dfrA12, dfrA15, dfrA27, dfra14, dfra21, dfrB4, dfrG, dfrK*	–	–	–	–	–	–
9	**Vancomycin**	Target modification	10	*vanA, VanB, vanHB, vanRC/RC4/TC, vanC2/vanC3, vanR, vanS, vanX*	–	–	–	–	–	–
10	**FCA**	Efflux / Enzymatic inactivation / Target protection	18	*catB3/8/9/Q, cat, catIII, cat(pC221), floR, cmlA5, QnrB4, QnrS1/S3/S5, qnrD, qnrS2, mdtH, pmrA, qepA_1_2, qacA_B*	–	–	–	–	–	–
11	**Fluoroquinolone / Quinolone**	Efflux / Target modification	3	*abaQ, qepA, norB*	4	*gyrA, parC, parE, gyrB*	–	–	–	–
12	**Phenicol / Chloramphenicol**	Efflux / Enzymatic inactivation	4	*cat, catB, floR, chloramphenicol exporter*	–	–	2	*floR, cat2*	–	–
13	**Polymyxin**	Target modification	3	*arnA, icr–Mo, mcr–5*	3	*arnA, phoP, PMrA*	–	–	–	–
14	**Rifamycin**	Enzymatic inactivation	2	*ADP–ribosylating transferase, rifampin monooxygenase*	1	*rpoB*	–	–	–	–
15	**Fosfomycin / Fosmidomycin**	Enzymatic inactivation / Efflux	3	*fosX, rosA, rosB*	1	*murA*	–	–	–	–
16	**Bacitracin**	Target modification	2	*bacA, bcrA*	–	–	–	–	–	–
17	**Others / Unclassified**	Various / Unknown	11	*sat–4, mcr–1, arr–2/3, kasugamycin ksgA, cAMP–regulatory protein, CpxR, ArlR, DNA–binding protein, sdiA*	–	–	–	–	–	–
	**Total**		**326**		**33**		**17**		**3**	

Note: *ARG classes are restricted to the core resistome of L. J. Li et al. (2024). Gene subtypes are shown in italic. – = not detected; n* = number of gene subtypes detected. ARG = antibiotic resistance gene; FCA = fluoroquinolone–chloramphenicol–aminoglycoside; MLSB = macrolide–lincosamide–streptogramin B; MGE = mobile genetic element; DHPS = dihydropteroate synthase; DHFR = dihydrofolate reductase.
